# Supplementary material for: Modeling glioblastoma heterogeneity as a dynamic network of cell states
Source: Mol Syst Biol. 2021 Sep 16;17(9):e10105. doi: 10.15252/msb.202010105 (PMC8444284; doi:10.15252/msb.202010105)
Supplement: Supplementary file 6 — Source Data for Figure 5 [file MSB-17-e10105-s004.zip › Figure5A_sourcedata/GSEA_3071/hallmarks_stateI.GseaPreranked.1623742531165/HALLMARK_UNFOLDED_PROTEIN_RESPONSE.html]

Details for gene set HALLMARK\_UNFOLDED\_PROTEIN\_RESPONSE[GSEA]

|  || Dataset | state13071 |
| Phenotype | NoPhenotypeAvailable |
| Upregulated in class | na\_pos |
| GeneSet | HALLMARK\_UNFOLDED\_PROTEIN\_RESPONSE |
| Enrichment Score (ES) | 0.32669812 |
| Normalized Enrichment Score (NES) | 1.6959859 |
| Nominal p-value | 0.0 |
| FDR q-value | 0.024121774 |
| FWER p-Value | 0.219 |
Table: GSEA Results Summary

  

Fig 1: Enrichment plot: HALLMARK\_UNFOLDED\_PROTEIN\_RESPONSE      
 Profile of the Running ES Score & Positions of GeneSet Members on the Rank Ordered List

  

| PROBE | GENE SYMBOL | GENE\_TITLE | RANK IN GENE LIST | RANK METRIC SCORE | RUNNING ES | CORE ENRICHMENT || 1 | KDELR3 |  |  | 136 | 0.951 | 0.0031 | Yes |
| 2 | NABP1 |  |  | 154 | 0.907 | 0.0387 | Yes |
| 3 | XBP1 |  |  | 215 | 0.797 | 0.0569 | Yes |
| 4 | CEBPB |  |  | 222 | 0.782 | 0.0900 | Yes |
| 5 | XPOT |  |  | 314 | 0.658 | 0.0931 | Yes |
| 6 | HSPA5 |  |  | 337 | 0.638 | 0.1152 | Yes |
| 7 | PSAT1 |  |  | 338 | 0.638 | 0.1437 | Yes |
| 8 | SHC1 |  |  | 361 | 0.624 | 0.1652 | Yes |
| 9 | SEC31A |  |  | 367 | 0.619 | 0.1913 | Yes |
| 10 | FKBP14 |  |  | 418 | 0.575 | 0.2026 | Yes |
| 11 | HERPUD1 |  |  | 436 | 0.561 | 0.2227 | Yes |
| 12 | EXOC2 |  |  | 507 | 0.503 | 0.2249 | Yes |
| 13 | SEC11A |  |  | 518 | 0.496 | 0.2442 | Yes |
| 14 | DNAJC3 |  |  | 533 | 0.489 | 0.2619 | Yes |
| 15 | MTHFD2 |  |  | 588 | 0.459 | 0.2668 | Yes |
| 16 | DNAJB9 |  |  | 605 | 0.448 | 0.2821 | Yes |
| 17 | WIPI1 |  |  | 620 | 0.443 | 0.2979 | Yes |
| 18 | DDX10 |  |  | 761 | 0.394 | 0.2750 | Yes |
| 19 | SSR1 |  |  | 795 | 0.384 | 0.2826 | Yes |
| 20 | HSP90B1 |  |  | 798 | 0.382 | 0.2991 | Yes |
| 21 | DDIT4 |  |  | 865 | 0.361 | 0.2961 | Yes |
| 22 | SPCS3 |  |  | 927 | 0.343 | 0.2938 | Yes |
| 23 | HYOU1 |  |  | 961 | 0.336 | 0.2993 | Yes |
| 24 | SRPRA |  |  | 975 | 0.333 | 0.3104 | Yes |
| 25 | SLC30A5 |  |  | 1020 | 0.323 | 0.3121 | Yes |
| 26 | SLC7A5 |  |  | 1084 | 0.310 | 0.3077 | Yes |
| 27 | YIF1A |  |  | 1088 | 0.310 | 0.3207 | Yes |
| 28 | ATF4 |  |  | 1115 | 0.304 | 0.3267 | Yes |
| 29 | SLC1A4 |  |  | 1184 | 0.292 | 0.3201 | No |
| 30 | ERN1 |  |  | 1297 | 0.269 | 0.2997 | No |
| 31 | TTC37 |  |  | 1368 | 0.257 | 0.2909 | No |
| 32 | EIF4G1 |  |  | 1433 | -0.251 | 0.2837 | No |
| 33 | NOP14 |  |  | 1559 | -0.263 | 0.2593 | No |
| 34 | VEGFA |  |  | 1644 | -0.273 | 0.2472 | No |
| 35 | EXOSC4 |  |  | 1723 | -0.283 | 0.2373 | No |
| 36 | EXOSC9 |  |  | 1777 | -0.291 | 0.2349 | No |
| 37 | SPCS1 |  |  | 1887 | -0.303 | 0.2169 | No |
| 38 | EIF4E |  |  | 1900 | -0.305 | 0.2271 | No |
| 39 | EIF4A1 |  |  | 2002 | -0.317 | 0.2121 | No |
| 40 | KHSRP |  |  | 2027 | -0.322 | 0.2195 | No |
| 41 | DCP1A |  |  | 2149 | -0.343 | 0.1998 | No |
| 42 | EXOSC5 |  |  | 2297 | -0.372 | 0.1739 | No |
| 43 | FUS |  |  | 2300 | -0.373 | 0.1900 | No |
| 44 | CKS1B |  |  | 2332 | -0.380 | 0.1980 | No |
| 45 | NOP56 |  |  | 2333 | -0.380 | 0.2150 | No |
| 46 | DKC1 |  |  | 2365 | -0.386 | 0.2232 | No |
| 47 | RRP9 |  |  | 2373 | -0.388 | 0.2385 | No |
| 48 | EXOSC2 |  |  | 2400 | -0.392 | 0.2485 | No |
| 49 | EIF2S1 |  |  | 2638 | -0.450 | 0.2001 | No |
| 50 | NHP2 |  |  | 2684 | -0.468 | 0.2080 | No |
| 51 | LSM4 |  |  | 3115 | -0.688 | 0.1144 | No |
Table: GSEA details [plain text format]

  

Fig 2: HALLMARK\_UNFOLDED\_PROTEIN\_RESPONSE: Random ES distribution      
 Gene set null distribution of ES for **HALLMARK\_UNFOLDED\_PROTEIN\_RESPONSE**
